# Homœopathy and Regular Medicine

**Published:** 1871-09

**Authors:** E. V. Stoddard

**Affiliations:** Rochester, N. Y.


### Homœopathy and Regular Medicine.

Rochester, N. Y., September, 1871.

*To the Editor of the Buffalo Medical and Surgical Journal.*

My Dear Sir,—

In the last number of the *Buffalo Medical and Surgical Journal*, I noticed an article, entitled " Homœopathy and Regular Medicine." This production bears the signature of a young member of your County Society. Upon the closing page of the Journal, is an editorial note, calling attention to this article, and offering to any of your readers an opportunity for replying to, or correcting its statements *if* they deem it *necessary*. I trust that they all feel as I do in regard to it. My sentiments are expressed very fully in *Hosea 7th chap. 8th verse*, " Ephraim is a cake not turned"; and again in *Hosea, 4th chap. 17th verse*, " Ephraim is joined to his *idols ; let him alone.*"

Accompanying please find a copy of our revised by-laws, &c., of Monroe County Medical Society, just issued.

I am, with esteem, very sincerely, yours,

E. V. Stoddard.

